# Mortality Rate of Sepsis Patients in the Intensive Care Unit: The Prognostic Role of Ejection Fraction and Procalcitonin

**DOI:** 10.31661/gmj.v10i0.2044

**Published:** 2021-06-22

**Authors:** Mohammad Esmaeil Hejazi, Ali Hossein Samadi-Takaldani, Abdolmohammad Ranjbar, Mohammad Negaresh, Yasin Hejazi

**Affiliations:** ^1^Department of Internal Medicine, Tabriz University of Medical Sciences, Tabriz, Iran; ^2^Department of Internal Medicine, Ardabil University of Medical Sciences, Ardabil, Iran; ^3^Department of Cardiology, Tabriz University of Medical Sciences, Tabriz, Iran

**Keywords:** Procalcitonin;, Ejection Fraction;, Intensive Care Unit

## Abstract

Sepsis is the second leading cause of death in the intensive care unit (ICU) and is one of the important causes of death for all hospitalized patients [1]. Evidence revealed procalcitonin as the critical risk factor for determining the prognosis of septic patients [2,3].Also, new studies indicated that diastolic dysfunction and low ejection fraction (EF) were identified as risk factors for death in septic patients [4]. Indeed, septic patients with lower EF had higher mortality rates than other septic patients [5]. Hence, in the pilot study, we determine the mortality rate of hospitalized patients in our clinic during 2020. Besides, EF was evaluated via echocardiography, and also serum PCT was measured on the first day of admission to ICU. Our results indicate that 35 % and 65 % of patients were expired and discharged, respectively. The association between EF and mortality is shown in Figure-1. There were no significant differences between EF and mortally among studied patients (P=0.79). The mean PCT in expired patients was 7.67 ±5.52 ng/ml, while in the discharged patients was 4.21±3.1 ng/ml. On the other hand, although the mean PCT level in the expired patients was higher than those discharged, this difference was not significant. Our study revealed that although PCT and EF statistically were not different in expired patients compared to those discharged, both PCT and EF could be considered important prognostic factors for mortality among sepsis patients in the ICU. However, more studies with larger sample sizes and more parameters for the determination of EF and PCT are recommended.

## Conflict of Interest

The authors declare there was no any conflict of interest.

**Figure 1:**
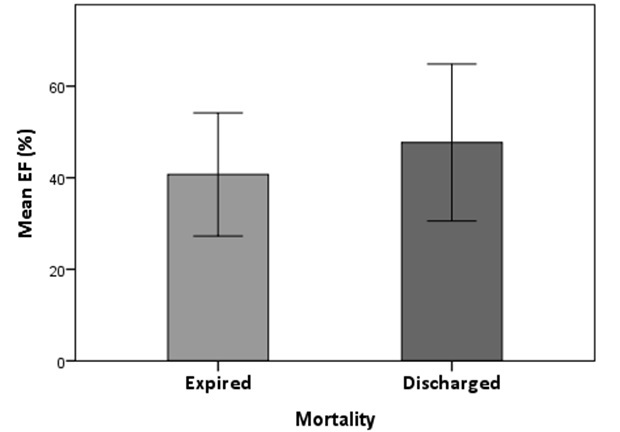
The association between EF and mortality of septic patients
